# Predictive Accuracy of Glasgow Coma Scale and Pupillary Data on Presence of Traumatic Brain Injury

**DOI:** 10.3390/jcm15020697

**Published:** 2026-01-15

**Authors:** Diana Schüller, Arasch Wafaisade, Rolf Lefering, Filippo Migliorini, Eftychios Bolierakis, Matthias Weuster, Yusuke Kubo, Matthias Fröhlich, Arne Driessen

**Affiliations:** 1 Department of Orthopaedics, Trauma and Reconstructive Surgery, University Hospital RWTH Aachen, RWTH Aachen University, 52074 Aachen, Germany; dianaschller@gmail.com (D.S.); migliorini.md@gmail.com (F.M.); ebolierakis@ukaachen.de (E.B.); arne.driessen@luisenhospital.de (A.D.); 2 Department of Orthopaedic Surgery, Trauma Surgery and Sports Traumatology, University of Witten/Herdecke, Cologne-Merheim Medical Centre (CMMC), 51109 Cologne, Germany; wafaisadea@kliniken-koeln.de; 3 Institute for Research in Operative Medicine (IFOM), University of Witten/Herdecke, 51109 Cologne, Germany; rolf.lefering@uni-wh.de; 4 Department of Trauma-, Hand- and Plastic Surgery, Diako Hospital, 24939 Flensburg, Germany; matthias.weuster@diako.de; 5 Department of Anatomy and Cell Biology, RWTH Aachen University, 52074 Aachen, Germany; y.kubokku@gmail.com; 6 Department of Orthopaedic Surgery, Graduate School of Medical Science, Kyusho University, 3-1-1 Maidashi, Higashi–ku, Fukuoka 812-8582, Japan; 7 Department of Trauma and Orthopaedic Surgery, Luisenhospital, 52064 Aachen, Germany; 8 Committee on Emergency Medicine, Intensive Care and Trauma Management (Sektion NIS), The German Trauma Society (DGU), 10623 Berlin, Germany

**Keywords:** traumatic brain injury, prediction outcome, Glasgow Coma Scale, pupil reactivity, pupil size

## Abstract

**Background/Objectives:** The GCS is widely used to assess a patient’s level of consciousness after trauma. Although not a diagnostic tool for traumatic brain injury (TBI), prehospital clinicians frequently rely on GCS findings—along with pupil exam, mechanism of injury, and clinical presentation, to estimate the likelihood that TBI may be present before imaging is available. However, the GCS has known limitations and fails to identify a significant proportion of TBI patients. This study aimed to evaluate the association between GCS scores and the presence of TBI, and whether additional clinical variables improve its discriminatory value. **Methods:** This retrospective cohort study analyzed data from trauma patients registered in the TraumaRegister DGU^®^ between 2015 and 2017. TBI was defined as a head injury with an Abbreviated Injury Scale (AISHead) score of ≥2. Inclusion criteria consisted of trauma team activations with a maximum AIS ≥ 3 and/or the need for intensive care. Prognostic values were assessed using multivariable logistic regression analysis. **Results:** 40,216 patients were included of which 17,205 (42.8%) were diagnosed with TBI and 23,011 (57.2%) were non-TBI patients. In the TBI group, 36.4% (n = 6216) presented with an initial GCS of 15 prehospitally. 17.8% (n = 3059) of TBI patients had anisocoric or bilaterally dilated pupils, 22.1% (n = 3799) had sluggish or fixed light reactivity and 17% (n = 2934) had no motoric response in Eppendorf-Cologne Scale (ECS) motor component. GCS score by itself showed better TBI prediction value than pupil size or reactivity or motor component alone. Nevertheless, substantial misclassification was observed when using GCS alone: 25.7% of patients with a normal GCS (15) had TBI (AIS Head ≥ 2), while 19.1% of patients with GCS 3 had no TBI. In the non-TBI group, 2.7% (n = 622) had a GCS of 3, 2.9% (n = 685) had anisocoric or bilaterally dilated pupils, 4.2% (n = 960) had sluggish or fixed light reactivity and 3.3% (n = 751) had no motoric response. Even at the lowest GCS score of 3, 19.1% of patients did not have TBI, while a normal GCS of 15 still included 25.7% of patients with TBI. **Conclusions:** The expanded model combining GCS with pupillary assessment and the ECS motor component demonstrated superior performance in prehospital TBI detection compared with the GCS alone. Implementing an extended GCS incorporating pupillary and ECS assessment may facilitate earlier recognition of TBI and support timely triage decisions; however, potential effects on patient outcomes require confirmation in prospective studies.

## 1. Introduction

Traumatic brain injury (TBI) is damage to the brain caused by external mechanical force, which can lead to permanent or temporary impairment of cognitive, psychosocial, and physical functions, as well as a reduced or altered state of consciousness [1]. Severe traumatic brain injury (TBI) affects approximately 10 million people globally each year [2]. TBI is the leading cause of death, disability, and traumatic mortality [3]. Over recent decades, advancements in interdisciplinary collaborations have significantly improved TBI treatment [4]. The Glasgow Coma Scale (GCS) has greatly enhanced communication among medical staff by providing a practical method to describe a patient’s condition and level of consciousness in clinical settings [5].

The GCS, developed in 1974 by Teasdale and Jennett, was designed to standardize the evaluation of a patient’s level of consciousness following head trauma [5]. It includes three components that can be assessed by even inexperienced personnel in prehospital settings without additional diagnostic tools. Beyond the initial prehospital evaluation, the GCS is used clinically to monitor changes in neurological function in patients with suspected or confirmed TBI. Clinical assessments often include the GCS alongside pupil examination, CT imaging, and continuous monitoring of blood pressure and pulse oximetry [6]. Early recognition of TBI severity and timely intervention are known to be associated with improved patient outcomes, as about half of all fatal brain injuries occur within the first hour after trauma [7]. Rapid transport to designated trauma centers has been shown to reduce mortality in TBI patients [8]. Accurate prehospital assessment is crucial due to its substantial impact on prognosis. However, detecting TBI in prehospital settings remains challenging [9]. Cooke et al. demonstrated that severe brain injury might still occur in patients presenting with high GCS scores [10]. Since the GCS primarily evaluates consciousness, it may not identify patients with initially intact sensory perception who have sustained severe TBI, a phenomenon known as the “talk and die” syndrome [11]. Conversely, various traumatic or medical factors, including hypotension, sedation, hypoglycemia, hemorrhage, or substance intoxication, can result in misleadingly low GCS scores [12,13].

Due to the various reasons for loss of consciousness apart from TBI, especially in multiple-injured patients, only a medium correlation was found between the GCS and the prediction of severe TBI [12]. Studies have compared the different GCS components to their predictive value of TBI, with results indicating the highest predictive accuracy for the motor component and only minimal improvement with the addition of verbal and eye components [14,15,16]. To improve predictive accuracy, more specifically to TBI, different risk factors associated with poor outcome have been identified in the past, such as age, systemic hypotension, intracranial hypertension, or absence of pupillary light reflex [17]. Aside from GCS, pupillary assessment is also a commonly used neurological screening tool [18].

A review comparing different literature on the predictive ability of the GCS concluded that this ability is improved if other clinical variables are added to the GCS, for example, pupillary response or age [19]. A study by Hoffman and colleagues showed that for predicting the outcome of brain-injured patients, pupil reactivity and ECS motor component exceed the GCS alone, demonstrating the importance of pupillary assessment [20]. Brennan et al. also combined GCS scores and pupil scores into a newly formed GCS-P score, superior to GCS in predicting patient mortality [21].

When comparing GCS components and pupil evaluations, pupil size and reactivity have demonstrated superior predictive accuracy for TBI. Specifically, pupil size outperforms the GCS motor component, while pupil reactivity surpasses both the GCS motor and eye components. Notably, the GCS verbal response exhibits the highest predictive accuracy among GCS components [16]. These findings suggest that incorporating pupil reactivity and size assessments may enhance the predictive value for TBI presence and related mortality.

Furthermore, the GCS alone underperforms in predicting mortality compared to assessments focusing on pupil reactivity and the GCS motor component [16]. A comprehensive study utilizing data from the TraumaRegister DGU^®^ (1993–2010) evaluated the predictive accuracy of the GCS against a modified GCS motor component and the Eppendorf Cologne Scale (ECS), which incorporates pupil reactivity and size. The ECS demonstrated a significantly higher predictive value [3].

In the present study, substantial overlap existed between GCS scores and anatomical TBI. This degree of misclassification illustrates the limitations of relying on GCS alone. It was aimed to assess whether the addition of other clinical variables to the GCS was independently associated with TBI and could enhance its diagnostic utility in the prehospital setting. Specifically, we assessed the combined predictive value of the GCS with pupil size, pupil reactivity, and the Eppendorf-Cologne Scale (ECS) motor component. TBI diagnosis was based on the Abbreviated Injury Scale (AISHead) score ≥ 2, as recorded in the TraumaRegister DGU^®^ database.

## 2. Materials and Methods

### 2.1. TraumaRegister DGU^®^

The TraumaRegister DGU^®^ of the German Trauma Society (Deutsche Gesellschaft für Unfallchirurgie, DGU) was founded in 1993. The aim of this multicentre database is to provide pseudononymized and standardized documentation of severely injured patients. The data are collected prospectively in four consecutive time phases from the site of the accident until discharge from the hospital: (A) prehospital phase, (B) emergency room and initial surgery, (C) intensive care unit, and (D) discharge. The documentation includes detailed information on demographics, injury patterns, comorbidities, pre- and in-hospital management, progression in the intensive care unit, and relevant laboratory findings, including data on transfusion and the outcome of each individual patient. The inclusion criterion is hospital admission via the emergency room with subsequent ICU/ICM or arriving at the hospital with vital signs and death before admission to the ICU. The infrastructure for documentation, data management, and data analysis is provided by the AUC—Academy for Trauma Surgery (AUC—Akademie der Unfallchirurgie GmbH), a company affiliated with the German Trauma Society. The scientific leadership is provided by the Committee on Emergency Medicine, Intensive Care and Trauma Management (Sektion NIS) of the German Trauma Society. The participating hospitals submit their data pseudonymized into a central database via a web-based application. Scientific data analysis is approved according to a peer review procedure established by Sektion NIS. The participating hospitals are primarily located in Germany (90%), but an increasing number of hospitals in other countries contribute data as well (such as Austria, Belgium, China, Finland, Luxembourg, Slovenia, Switzerland, the Netherlands, and the United Arab Emirates). Currently, over 25,000 cases from almost 700 hospitals are entered into the database per year. Participation in the Trauma Register DGU^®^ is voluntary. For hospitals associated with the TraumaNetzwerk DGU^®^, however, the entry of at least one basic data set is obligatory for reasons of quality assurance. All injuries are coded using the Abbreviated Injury Scale (AIS) [2]. Scientific data analysis is approved according to a peer review procedure laid down in the publication guideline of TR-DGU^®^.

The present study is in line with the publication guidelines of the TraumaRegister DGU^®^ and is registered as TR-DGU project ID 2019–004.

### 2.2. Data Analysis

Datasets of trauma patients entered into the TraumaRegister DGU^®^ (TR-DGU) between 2015 and 2017 were included for analysis.

**Figure 1:**
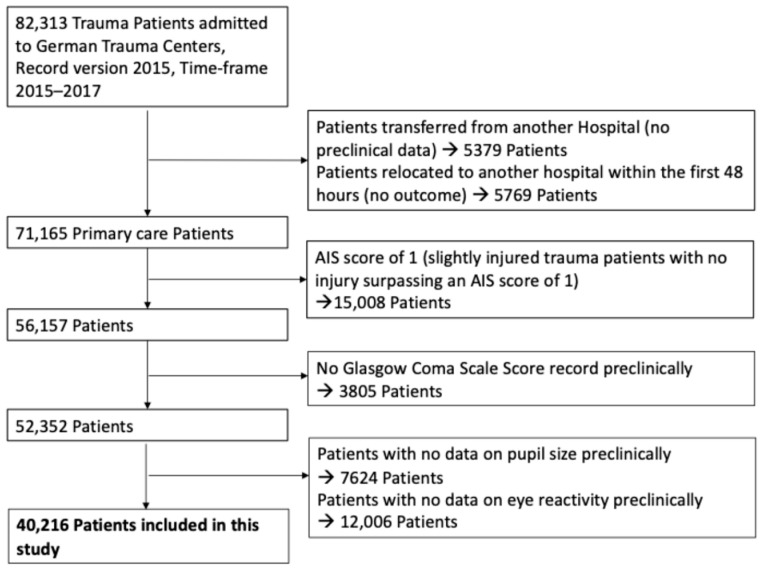
Study diagram detailing selection of patients for this study (Exclusion categories are not mutually exclusive).

Traumatic brain injury (TBI) was defined as a head injury with a maximum Abbreviated Injury Scale for the head region (AISHead) score of ≥2, encompassing moderate to severe TBI cases. Patient selection and sample size are illustrated in Figure 1. All neurological assessments, including Glasgow Coma Scale (GCS) score, ECS motor response, pupil size, and pupil reactivity, were obtained in the prehospital phase, as documented in the TraumaRegister DGU^®^. These measurements occurred prior to hospital arrival and formal diagnosis of TBI, ensuring that all predictive variables reflect the patient’s condition at first clinical contact. For the purposes of this study, “misclassified” TBI cases refer to patients who exhibited clinical signs suggestive of TBI in the prehospital setting (e.g., low GCS, abnormal pupils, suspicious mechanism) but were ultimately found not to have TBI, as defined by an AISHead score < 2. The AISHead score is determined post hoc based on imaging and in-hospital evaluation. AISHead codes were derived from ICD-based injury codes documented in the TraumaRegister DGU^®^. This operational definition enabled consistent categorization across a large retrospective cohort.

Inclusion criteria were as follows:

- Primary admission to a German hospital participating in the TraumaRegister DGU^®^.
- Activation of the trauma team upon arrival.
- Maximum Abbreviated Injury Scale (MAIS, the highest AIS score of all injuries of a person) score ≥ 3, or MAIS = 2 with admission to intensive care.
- Availability of complete prehospital data for Glasgow Coma Scale (GCS), pupil size, and pupil reactivity.

Exclusion Criteria were as follows:

- Missing or incomplete prehospital documentation of GCS, pupil size, or pupil reactivity.
- Secondary transfers from another facility.
- Relocation to another facility within the first 48 h (no outcome)
- Slightly injured trauma patients with no injury surpassing an AIS score of 1.
- Patients lacking prehospital data on the Glasgow Coma Scale (GCS) or pupillary parameters were excluded from analysis, as these were central to the study objectives. The exact numbers are presented in Figure 1. All analyses were performed using a complete-case approach; no imputation of missing data was conducted.

The primary outcome of this analysis was the presence of traumatic brain injury (TBI), defined operationally as a head injury with an Abbreviated Injury Scale (AISHead) score of ≥2, as recorded in the Trauma Register DGU^®^. Secondary outcomes included the predictive accuracy of individual clinical variables (GCS score, ECS motor component, pupil size, pupil reactivity) for detecting TBI, the additive predictive value when combining GCS with pupil and motor assessments and the odds ratio of each variable for association with TBI, assessed through multivariable logistic regression.

### 2.3. Abbreviated Injury Scale (AIS)

The AIS is used to categorize injuries on a six-point scale, with scores ranging from 1 (minor injury) to 6 (fatal injury):

- AISHead = 1: Minor head injury, such as superficial contusions or mild concussion without loss of consciousness.
- AISHead = 2: Moderate head injury, including brief loss of consciousness, small intracranial hemorrhages, or minor skull fractures without brain involvement.
- AISHead = 3: Serious head injury with significant anatomical damage and life-threatening potential, such as larger intracranial hemorrhages or skull fractures with brain involvement.
- AISHead = 4: Severe head injury with extensive damage and high morbidity/mortality risk, for example, large intracranial hematomas requiring surgery, severe diffuse axonal injury, or extensive skull fractures with brain protrusion.
- AISHead = 5: Critical injury with a high mortality risk, including massive intracranial hemorrhages with brain herniation, extensive brain swelling, or severe penetrating trauma.
- AISHead = 6: Unsurvivable injury characterized by catastrophic damage incompatible with life, such as catastrophic penetrating head trauma.

### 2.4. Glasgow Coma Scale (GCS)

The GCS evaluates a patient’s level of consciousness through three components: motor response, verbal communication, and eye-opening. Severity of TBI is classified as follows (Table 1 [3]):

- Mild TBI: GCS 13–15 points
- Moderate TBI: GCS 9–12 points
- Severe TBI: GCS ≤ 8 points (with 3 points being the lowest possible score) [4]

### 2.5. ECS

The ECS is a prehospital neurological assessment tool developed as a simplified alternative to the GCS for predicting the presence and outcome of TBI in trauma patients. The ECS evaluates pupil reactivity, pupil size, and a simplified motor component derived from the GCS, classifying motor response into four subgroups (Table 1): normal (obeying commands), specific (localization or withdrawal to pain), nonspecific (abnormal flexion or extension), and none (no motor response) [3].

### 2.6. Pupillary Assessment

Based on previous analyses from the TR-DGU (ECS) [3], pupil evaluation includes both size and reactivity:

- Pupil Size:
  - Normal: Bilaterally non-dilated, non-constricted pupils.
  - Anisocoric: Unequal pupil sizes.
  - Bilaterally Dilated: Both pupils dilated beyond the normal range.
- Pupil Reactivity:
  - Brisk: Both pupils constrict promptly to light stimulation of either eye.
  - Sluggish: Delayed constriction response to light.
  - Fixed: No reaction to light in either eye.

### 2.7. Definition of TBI

TBI was defined as an AIS for the head region (AISHead) ≥ 2, including all intracranial injuries coded with a leading “1” in the AIS classification, while excluding cervical spine injuries. This threshold was chosen deliberately to capture the full clinical spectrum of brain injury severity and to demonstrate how impaired consciousness and abnormal pupillary findings correlate with increasing TBI severity.

## 3. Statistical Analysis

All data were analyzed using SPSS Statistics (Version 24, IBM Corp., Armonk, NY, USA). Categorical variables are reported as absolute numbers and percentages. Continuous variables are presented as means with standard deviations (SD) or, in the case of skewed distributions, as medians with interquartile ranges (IQR). Due to the large patient cohort, formal hypothesis testing was minimized to avoid statistical significance driven by trivial differences. Selected clinical variables (as presented in Table 2) were compared using the Chi-square test for categorical data and the Mann–Whitney U test for continuous variables. Multivariable logistic regression analysis was performed to assess the prognostic value of different variables associated with the presence of traumatic brain injury (TBI), defined as AISHead ≥ 2. The dependent variable in the model was TBI diagnosis (binary outcome: TBI vs. no TBI). The following independent variables were included based on clinical relevance and availability in prehospital settings: Glasgow Coma Scale (GCS) score (categorized: <15 vs. 15); ECS motor component (categorized: normal vs. abnormal); Pupil size (normal vs. anisocoric/dilated); Pupil reactivity (brisk vs. sluggish/fixed); Systolic blood pressure < 90 mmHg (indicator of shock); Mechanism of injury (e.g., low fall, high fall, traffic accident, penetrating trauma); Age (continuous). Variables with clinical plausibility, prior literature support, and low rates of missingness were prioritized. A *p*-value < 0.05 was considered statistically significant.

## 4. Results

A total of 40,216 patients were included in the analysis; patient selection is detailed in Figure 1. Among all patients included in the analysis, 17,205 (42.8%) were diagnosed with traumatic brain injury (TBI) (Table 2). Demographic data are detailed in Table 2. Patients in the TBI group were significantly older, with an average age of 54.8 years compared to 49.0 years in the non-TBI group (*p* < 0.001). A comparison of various clinical parameters between the TBI and non-TBI groups is provided in Table 2. Notably, the average length of hospital stay (LOS) was approximately one day shorter for the TBI group. Patients without TBI (n = 23,011) were less likely to require mechanical ventilation or resuscitation. No significant differences were observed between the groups regarding the use of anesthesia, chest tubes, blood transfusions, management of shock (systolic blood pressure < 90 mmHg), or volume therapy (Table 2).

### 4.1. Missing Data and Comparison of Included vs. Excluded Patients

To assess potential selection bias, we compared patients excluded due to missing prehospital neurological data (GCS or pupil parameters) with those included in the final analysis. The overall rate of missing GCS was 6.8%, with similar proportions among patients with and without TBI (6.4% vs. 7.1%), intubated and non-intubated patients (4.5% vs. 4.4%), and survivors and non-survivors (6.9% vs. 5.7%). Pupillary data were missing in approximately 13% of cases, again without major group differences. Excluded and included patients showed comparable characteristics in age (51.6 vs. 53.7 years), injury severity (ISS 17.4 vs. 17.0), TBI frequency (17.1% vs. 16.6%), intubation rate (14.7% vs. 14.4%), and mortality (16.9% in both groups). These findings suggest that missing data are unlikely to have introduced clinically relevant bias.

When examining trauma mechanisms, a lower percentage of TBI patients were involved in car accidents (16.0%, n = 2719 vs. 27.4%, n = 6273) or motorcycle accidents (8.2%, n = 1398 vs. 17.7%, n = 4056). Conversely, TBI was more frequently caused by low falls (34.1%, n = 5801) and bicycle accidents (12.9%, n = 2198).

### 4.2. Consciousness and Pupil Assessment

**Figure 2:**
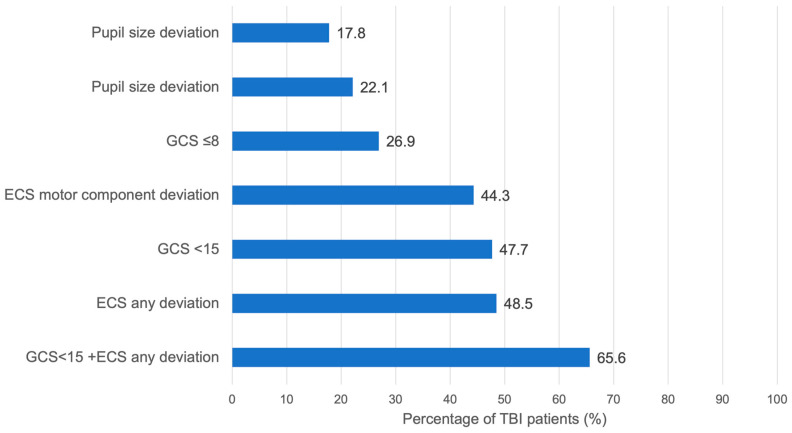
Sensitivity of specific early symptoms in patients with TBI (AIS_Head_ ≥ 2).

Overall, 61% (n = 24,384) of patients presented with a prehospital Glasgow Coma Scale (GCS) score of 15, indicating no verbal, motor, or eye-opening impairments. Within the TBI group, 36.4% (n = 6216) had an initial GCS of 15, whereas 15.3% (n = 2640) scored 3. In comparison, only 2.7% (n = 622) of non-TBI patients had a GCS of 3, while 78.8% (n = 18,123) scored 15. In total, 93% (n = 21,400) of the non-TBI group had a GCS of 12 or higher. The combined use of all three GCS components provided better TBI prediction than using pupil size, reactivity, or the ECS motor component alone (Figure 2). Nevertheless, there was substantial overlap between prehospital GCS assessment and anatomical TBI severity. in the overall cohort, 25.7% (n = 6261) of patients with a prehospital GCS of 15 were subsequently diagnosed with TBI (AIS Head ≥ 2), highlighting that a normal GCS alone does not exclude TBI. Also, patients presenting with the lowest possible GCS score (3), 19.1% had no relevant TBI (AIS Head < 2). Conversely, within the confirmed TBI group, 36.4% of patients presented with an initial GCS of 15, indicating that more than one-third of patients with anatomical evidence of TBI were fully conscious at the scene.

Normal pupil reactivity was found in 95.8% (n = 22,051) of non-TBI patients. Notably, nearly 80% of TBI patients (n = 13,406) also had normal pupil reactivity. Anisocoria was present in 10.7% (n = 1847) of TBI patients, followed by bilateral dilation in 7.1% (n = 1212). Despite these findings, about 80% of TBI patients had normal pupil size (Table 3).

Among TBI patients, 82.2% (n = 14,146) had normal pupil size, while 17.8% (n = 3059) presented with anisocoria or bilaterally dilated pupils. In the non-TBI group, anisocoria or bilateral dilation was observed in 2.9% (n = 685), and sluggish or fixed light reactions occurred in 4.2% (n = 960). In the TBI group, 77.9% (n = 13,406) exhibited normal light reactivity, which is 740 fewer than those with normal pupil size. Sluggish or fixed reactivity was noted in 22.1% (n = 3799) of TBI patients. Deterioration of the motor component of the GCS was associated with a higher incidence of TBI (Table 4); 17% (n = 2934) of TBI patients showed no motor response, although 3% (n = 751) of patients without motor response did not have TBI.

### 4.3. Prediction of TBI

Logistic regression analysis revealed that a GCS below 15 quadrupled the likelihood of TBI. Impairments in pupil size and reactivity increased the probability by 2.0 to 2.5 times, enhancing the predictive value of the GCS. A low fall (<3 m) doubled the risk of TBI, whereas hypotension (systolic blood pressure < 90 mmHg) and penetrating trauma significantly decreased TBI probability (odds ratio 0.31–0.43).

Prehospital diagnosis accuracy for patients with TBI (AIS Head ≥ 2) varied by clinical severity on admission: 43.8% (n = 3757) of patients with severe TBI (GCS ≤ 8), 31.9% (n = 2732) with moderate TBI (GCS 9–12), and 14.2% (n = 1214) with mild TBI (GCS 13–15) were correctly identified prehospitally. Although 60.2% (n = 5880) of non-TBI cases were accurately predicted, 39.8% (n = 3886) were falsely classified as TBI, with 5.3% (n = 517) even misdiagnosed as severe TBI.

Four variables most significantly associated with TBI were identified: (1) GCS below 15, (2) abnormal pupil size, (3) impaired pupil reactivity, and (4) low fall mechanism. Patients with all five risk factors had a 10.7-fold increased probability of TBI compared to those without these factors (Table 5). Including these easily assessed variables in prehospital evaluations improved the model’s ability to discriminate TBI, suggesting a potential contribution to prehospital diagnostic accuracy, facilitating timely and appropriate interventions.

ROC (receiver operating characteristic) curve analysis was performed to assess the discriminatory performance of the multivariable model and selected predictors. The full multivariable model presented in Table 5 achieved an AUC (area under the curve) of 0.785 (95% CI: 0.781–0.790), reflecting good discriminatory ability for TBI (Table 6). Removing pupil-related variables slightly reduced the AUC to 0.777 (95% CI: 0.772–0.782), highlighting the incremental value of pupillary assessments. As an individual predictor, GCS < 15 demonstrated an AUC of 0.712 [95% CI: 0.707–0.717], while GCS ≤ 8 had an even lower discriminatory ability (AUC: 0.613 [95% CI: 0.607–0.619]). These results emphasize the utility of integrating multiple variables into prehospital assessments to attempt improving the prehospital identification of TBI.

## 5. Discussion

This study aimed to evaluate whether the predictive accuracy of prehospital neurological assessment based on the GCS could be improved for identifying patients with traumatic brain injury TBI by incorporating additional variables. While the GCS is not a diagnostic test for TBI, it remains the primary neurological assessment tool available on site prior to imaging. Our study, therefore, focuses not on the diagnostic capacity of the GCS itself, but on its real-world discriminatory performance in a large, multicenter cohort, and whether additional simple variables improve this discrimination. Rapid and accurate identification of suspected TBI patients, followed by immediate transport to a trauma center with comprehensive treatment capabilities, is crucial; approximately half of patients with severe TBI succumb within the first two hours post-injury [2]. Although the GCS demonstrated the highest standalone predictive accuracy (Figure 2), a key finding of this study is the substantial misclassification associated with GCS alone, with more than one-quarter of GCS-15 patients having TBI and nearly one-fifth of GCS-3 patients having no TBI. These overlap rates provide the primary justification for evaluating supplementary neurological variables.

The analysis revealed that only two-thirds of TBI patients were directly admitted to trauma centers equipped with neurosurgical services. The higher mortality rate observed among the remaining third (Table 2) may be attributable to delayed access to definitive care, though further data on deceased patients and neurosurgical interventions are necessary to establish a significant correlation. Previous studies have underscored the detrimental impact of treatment delays on patient outcomes [4,5,6,7,8], and the decision to transport patients may be influenced by proximity to trauma centers, potentially explaining the higher TBI mortality rates in rural compared to urban areas [9]. These findings highlight the critical importance of prompt transportation to appropriate facilities.

Regression analysis (Table 5) indicates that adding variables—such as pupil size and reactivity, and the mechanism of injury (e.g., low falls or bicycle accident)—to the GCS may enhance clinical assessment of TBI likelihood compared to the GCS alone. ROC curve analysis (Table 6) confirms that the full multivariable model, which incorporates GCS, pupil assessments, and additional clinical variables, provides good discriminatory ability (AUC: 0.785). Excluding pupillary variables resulted in a slightly lower AUC of 0.777, indicating a modest but statistically significant improvement associated with their inclusion. The standalone performance of GCS < 15 was considerably lower (AUC: 0.712), underscoring the limitations of GCS alone. Although the inclusion of pupillary findings and ECS categories statistically improved model discrimination (AUC 0.785 vs. 0.777), this absolute increase is small and its direct clinical relevance must be interpreted with caution. The improvement indicates that these variables add information beyond the GCS alone, but the extent to which such a modest change may influence prehospital decision-making cannot be determined from this analysis. Rather than implying a direct clinical effect, these results highlight the potential value of a multimodal neurological assessment to improve the identification of patients at risk for TBI in prehospital settings, and reinforce the need for prospective studies to evaluate its practical implications for triage and clinical relevance.

As TBI remains a leading cause of traumatic deaths [10], improving prehospital diagnostic tools can reduce mortality rates. Existing clinical decision rules like the Canadian Head CT Rule and the New Orleans Criteria aim to identify patients at high risk for intracranial hemorrhage [11]; however, they are unsuitable for immediate prehospital application as they require patient monitoring over time [12]. In contrast, this study focuses on enabling rapid on-site TBI detection to facilitate appropriate triage decisions.

Motor vehicle collisions were the most common cause of TBI in the study population, followed by bicycle accidents. Low falls (<3 m) represented the second most common mechanism. Interestingly, low falls more than doubled the risk of TBI (OR 2.23), despite being considered a low-energy mechanism. This likely reflects the higher average age of the TBI cohort, as elderly patients are particularly prone to sustaining TBI even after seemingly minor trauma. Previous studies have reported similar findings, with same-level or low falls representing the leading cause of TBI-related hospitalization and death among patients older than 75 years [22,23,24]. Multiple age-related risk factors for low-level falls, like frailty, decreasing proprioception, osteoporosis, sensory decline, polypharmacy, and comorbidities like Parkinson’s disease, increase the risk of both falls and intracranial injury [25,26]. Consistent with these findings, most patients in the TBI group of our study are elderly (median 57y), and almost one third of TBI cases were caused by low falls. TBI-related hospitalization and mortality rates are highest among patients over 75 years old, correlating with the increased incidence of falls in this demographic [16]. Notably, nearly one-third of TBI patients in the study presented with a GCS of 15, which could have resulted in missed diagnoses if the GCS had been used as the sole assessment tool. This finding is partially explained by the study’s inclusion of moderate TBI cases, where patients may retain full consciousness.

However, substantial overlap between clinical and anatomical severity was also observed: 25.7% of patients with a GCS of 15 had TBI, whereas 19.1% of those with a GCS of 3 showed no structural TBI. These results highlight the limited discriminatory power of GCS alone and support the need for complementary neurological indicators to improve early recognition of TBI in the prehospital setting.

The GCS has recognized limitations in prehospital TBI prediction. Factors such as opioid overdose, alcohol or drug intoxication, hemorrhagic shock, hypoglycemia, or prehospital intubation can reduce responsiveness and compromise GCS reliability [19,20]. Brain injuries affecting speech centers can lower verbal response scores [27], and impaired brainstem reflexes may limit the scale’s effectiveness [27,28,29,30]. Studies have shown that non-neurosurgical clinicians frequently misapply the GCS [31,32], underscoring the need for improved training.

Previous research has primarily focused on the GCS’s association with functional outcomes [19,22,23,33,34,35,36], with fewer studies examining the combined predictive value of GCS components and additional variables [10,37,38]. One study using TraumaRegister DGU^®^ data found that the Reverse Shock Index multiplied by the GCS accurately predicted mortality risk but was unsuitable for admission decisions [36]. Among GCS components, the motor response consistently predicts outcomes most reliably [39], though recent evidence suggests the verbal response and pupil reactivity are superior in identifying TBI prehospitally [37]. Combining GCS with other variables, such as pupillary assessment and ECS motor function evaluation, enhances predictive accuracy [23].

Pupil size and reactivity, especially anisocoria and diminished light reflexes, have proven valuable for outcome prediction [10,37,40,41,42]. Abnormal pupillary responses correlate with elevated intracranial pressure and worse prognoses [18,43]. However, various factors—such as ambient light, substance use, or hypothermia—can affect pupillary assessment [15,20,44,45,46,47,48,49,50,51,52,53]. In this study, 83% of TBI patients had normal pupil size, while pupil reactivity abnormalities offered better predictive value. Nonetheless, abnormal pupillary findings also occurred in 38% of non-TBI patients, emphasizing the importance of comprehensive evaluation.

The ECS motor component closely followed pupil reactivity in predictive accuracy. Nearly 80% of patients without motor response belonged to the TBI group, although approximately 20% of non-TBI patients exhibited the same finding, likely due to unconsciousness from other causes. Data regarding paralysis or sedation were unavailable, highlighting a limitation. Despite these constraints, the combination of pupil size, pupil reactivity, and the GCS motor component outperformed the GCS alone. Considering the GCS’s complexity, simpler scales have shown comparable predictive power [54]; however, this study demonstrates that augmenting the GCS with additional variables can improve prehospital TBI detection, which may support more timely triage, although this study did not assess patient outcomes, and any potential clinical impact requires prospective validation.

### Limitations

This retrospective study is subject to inherent limitations, including potential documentation bias. Data variability, inaccuracies in record-keeping, and the fact that the data were not specifically collected for this analysis may affect the findings. Additionally, many TBI patients sustained multiple injuries, complicating the isolation of TBI-specific predictors. Patients managed outside the trauma system or those who died before hospital admission were excluded, potentially skewing results. While exclusion of patients with incomplete prehospital data could have introduced selection bias, our analyses demonstrated comparable demographics, injury severity, and outcomes between excluded and included cohorts, indicating that such bias is unlikely. Still, due to the substantial proportion of prehospital data missing, selection bias cannot be excluded and prospective studies with standardized neurological documentation are needed to fully overcome this limitation. Another key limitation is that patient outcomes, such as mortality or functional recovery, are not analyzed. Therefore, these results reflect diagnostic discrimination only, and implications for clinical outcomes remain to be clarified in future outcome-focused studies.

Neurosurgical intervention data were unavailable, and details regarding transfer times or the number of inter-facility transfers were not recorded. Incomplete documentation of substance use may have influenced GCS and pupillary assessments. Both assessments are subject to interrater variability, particularly when performed by less experienced personnel. External factors, such as lighting conditions at the scene, can also affect pupillary evaluations. Although the TR-DGU requires documentation of the first prehospital neurological assessment, the lack of precise timestamps does not rule out documentation after intubation or sedation completely; thus, confounding by pharmacologic interventions cannot be ruled out. Furthermore, the study did not stratify results by age, ethnicity, or socioeconomic status, limiting the generalizability of findings beyond Germany’s population (2015–2017). Also, the multivariate analysis primarily identifies associations between variables rather than establishing causal relationships or making predictive claims. The associations identified in this study are valuable for understanding patterns in the data; they do not provide a basis for predicting individual outcomes or establishing causal mechanisms. While AUC values reported here indicate good discriminatory performance, this analysis is retrospective and exploratory. Future prospective studies employing more advanced predictive models could help further clarify the extent to which the identified associations influence clinical outcomes. Finally, the study focused solely on prediction accuracy without directly correlating variables to patient outcomes like mortality or functional recovery. Future research should incorporate outcome measures to enhance the clinical utility of predictive models.

## 6. Conclusions

In this large, multicenter cohort, GCS alone showed substantial overlap between anatomical TBI and no TBI, underscoring its limited discriminatory ability in prehospital settings. Incorporating pupillary assessment and ECS motor response improved this discrimination modestly but significantly. These findings do not redefine the role of GCS as a diagnostic tool but rather underline the importance of a multimodal neurological assessment when estimating the likelihood of TBI before imaging is available, which may enhance early recognition of TBI and potentially improve patient outcomes by facilitating timely transport to appropriate trauma centers. However, the results should be interpreted as associative rather than causal and prospective and external validation studies are warranted to confirm these findings and to evaluate their clinical significance and applicability in real-world emergency care.

## Figures and Tables

**Table 1:** The Glasgow Coma Scale (adapted from [5]) and the ECS (Eppendorf-Cologne-Scale) Motor Component (adapted from [3]).

GCS	Points	0	1	2	3	4	5	6
	Motor Response		None	Abnormal Extension	Abnormal Flexion	Normal Flexion	Localizing	Obeying commands
	Verbal Response		None	Sounds	Words	Confused	Orientated	
	Eye Opening		None	To pain	To command	Spontaneous		
ECS motor component	Motor response	Normal	Specific	Nonspecific	None			

**Table 2:** Comparison of clinical and demographic parameters between TBI and non-TBI patients.

	TBI	No TBI	*p*-Value
No. of patients	17,205 (42.8%)	23,011 (57.2%)	
Males	11,600 (67.4%)	16,369 (71.1%)	<0.001
Mean Age (years)	54.8 (median 57)	49.0 (median: 50)	<0.001
Prehospital findings			
Heartrate (beats per minute)	89 (SD 22)	90 (SD 21)	<0.001
Systolic blood pressure sBP (mmHg)	136 (SD 35)	134 (SD 30)	<0.001
Shock (sBP ≤ 90 mmHg)	1289 (7.5%)	1571 (6.8%)	0.004
GCS score (median (IQR)	14 (8–15)	15 (15–15)	<0.001
GCS ≤ 8 (unconscious)	4626 (26.9%)	986 (4.3%)	<0.001
Volume therapy	14,292 (85.7%)	19,300 (86.1%)	0.28
Intubation	5234 (30.6%)	2452 (10.7%)	<0.001
Sedation ^#^	5186 (56.3%)	6657 (62.9%)	<0.001
Chest tube ^#^	278 (3.0%)	328 (3.1%)	0.77
CPR	715 (4.2%)	342 (1.5%)	<0.001
Time from accident to hospital admission (min)	63.7 (SD 28.5)	62.3 (SD 26.5)	0.003
Emergency Room			
Cranial computed tomography (cCT)	16,600 (96.9%)	19,835 (86.6%)	<0.001
pRBC transfusion	1095 (6.4%)	1393 (6.1%)	0.18
Length of stay in hospital (LOS), days	10 (5–19)	11 (6–19)	<0.001
Outcome			
Hospital mortality	2887 (16.8%)	999 (4.3%)	<0.001

^#^ not available in the reduced dataset; counts refer to cases with valid data only.

**Table 3:** Comparison of prehospital pupil reactivity and pupil size between TBI and non-TBI patients.

Pupil Reactivity	TBI	No TBI	Total
Brisk	13,406 (77.9%)	22,051 (95.8%)	35,457 (88.2%)
Sluggish	2276 (13.2%)	674 (2.9%)	2950 (7.3%)
Pupil size			
Normal	14,146 (82.2%)	22,326 (97%)	36,472 (90.7%)
Anisocoric	1847 (10.7%)	330 (1.4%)	2177 (5.4%)
Bilaterally dilated	1212 (7.1%)	355 (1.5%)	1567 (3.9%)
Total	17,205 (100%)	23,011 (100%)	40,216 (100%)

**Table 4:** Comparison of the frequency of different ECS motor component scores within TBI and non-TBI Patients.

ECS Motor Component	TBI	No TBI	Total
Normal	9587 (55.7%)	20,411 (88.7%)	29,998 (74.6%)
Specific	3811 (22.2%)	1702 (7.4%)	5513 (13.7%)
Nonspecific	873 (5.1%)	147 (0.6%)	1020 (2.5%)
None	2934 (17.0%)	751 (3.3%)	3685 (9.2%)

**Table 5:** Odds ratio of different factors predicting the occurrence of a TBI. This analysis is based on 37.150 patients with complete data (Nagelkerke’s R^2^ 0.324).

	Regression Coefficient	Standard Error	Odds Ratio (OR)	95% CI (Confidence Interval) for OR
Injury mechanism (reference: other)				
- car passenger - motor bike - bicycle - pedestrian - high fall (>3 m) - low fall (<3 m)	−0.27 −0.36 0.83 0.38 0.10 0.80	0.05 0.06 0.06 0.07 0.06 0.05	0.77 0.70 2.30 1.46 1.11 2.23	0.69–0.85 0.63–0.78 2.05–2.58 1.29–1.66 1.00–1.23 2.00–2.47
Age ≥ 70 years	0.27	0.30	1.31	1.23–1.39
Male gender	0.30	0.03	1.03	0.98–1.09
Shock (sBP ≤ 90 mmHg)	−0.61	0.05	0.54	0.49–0.60
Penetrating trauma	−1.00	0.08	0.37	0.31–0.43
Pupil reactivity not normal	0.75	0.05	2.11	1.91–2.33
Pupil size not normal	0.90	0.06	2.45	2.19–2.74
GCS < 15	1.36	0.03	3.88	3.64–4.14

**Table 6:** Discriminatory Performance of Multivariable Models and Key Predictor Combinations for TBI Diagnosis.

Model	AUC (95% CI)
Full multivariable model (Table 5)	0.785 (95% CI: 0.781–0.790)
Full model without pupil variables	0.777 (95% CI: 0.772–0.782)
GCS < 15	0.712 (95% CI: 0.707–0.717)
GCS ≤ 8	0.613 (95% CI: 0.607–0.619)

## Data Availability

The original contributions presented in this study are included in the article. Further inquiries can be directed to the corresponding author.

## Abbreviations

AIS—Abbreviated Injury Scale; AISHead—Head region of the Abbreviated Injury Scale; AUC—Akademie der Unfallchirurgie GmbH; AUC—Area Under the Curve; BP—blood pressure; CI—Confidence Interval; CT—computer tomography; cCT—cranial computed tomography; DGU—Deutsche Gesellschaft für Unfallchirurgie (German Trauma Society); ECS—Eppendorf-Cologne Scale; ED—emergency department; GCS—Glasgow Coma Scale; GCS-MC—GCS motor component; ICP—intracranial pressure; ICU—intensive care unit; IQR—Interquartile Range; ISS—Injury Severity Score; LOS—length of stay in hospital; MAIS—Maximum Abbreviated Injury Scale; MRI—magnetic resonance imaging; OR—Odds Ratio; PR—pupil reactivity; pRBC’s—packed red blood cells; PS—pupil size; ROC—Receiver Operating Characteristic; SD—standard deviation; Sektion NIS—Committee on Emergency Medicine, Intensive Care and Trauma Management; TBI—traumatic brain injury; TT—transportation time; TR-DGU—TraumaRegister DGU^®^.
